# Use of ready-made spectacles in school eye health programmes

**Published:** 2017-08-07

**Authors:** Priya Morjaria

**Affiliations:** 1 Research Fellow and Public Health Optometrist: International Centre for Eye Health, London, UK.

**Ready-made spectacles are suitable for a high proportion of children with refractive errors – but not everyone can benefit.**

The most common means of correcting refractive errors is with spectacles. Spectacles are prescribed and dispensed with corrective lenses that give the best visual acuity and are comfortable. Custom-made spectacles (i.e., made up for each individual) are more expensive, but they are essential in some cases, i.e., when a person requires astigmatic correction or needs different power lenses in each eye (anisometropia).

A standard way to report refractive error is to use the ‘spherical equivalent’, which is calculated as the sphere plus half the cylinder, in dioptres (for example, the spherical equivalent for a refractive error of +2.0D with a −1.0D cylinder is 2 + (−1.0/2) = 1.5D). In children who have no or low astigmatism, and only a small difference between the left and right eyes, their refractive error can be corrected using a pair of ready-made spectacles: low cost, high quality spectacles that have been pre-fitted with pairs of lenses of the same spherical equivalent.

## Advantages and disadvantages

The advantage of ready-made spectacles is that they are less expensive, can be dispensed immediately in schools or clinics, and require less time to dispense.

The drawback to ready-made spectacles is that it requires a large inventory of frames in different sizes, colours and shapes, each with a range of power lenses. They are only suitable if the prescription in both eyes is the same and lenses are seldom available in powers of over +/−3.5 D. That said, evidence from studies in Cambodia, China and India indicate that 70–90% of children with uncorrected refractive errors could benefit from ready-made spectacles.^1^,^2^,^3^

2.5 New Vision Generation, an Essilor Group initiative, has produced a range of spectacles called ‘Ready-to-Clip’ that allows on-the-spot delivery. The lenses, which are interchangeable between right and left, are clipped into the person's chosen frame according to their individual prescription. Lenses of different powers can be used in each eye, which means that some children with anisometropia can also benefit. Inventory is also reduced.

**Figure 1:**
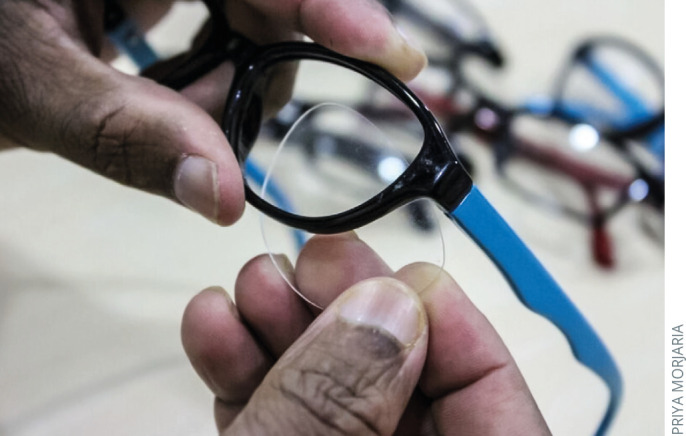
Lens being clipped into a ‘clip-and-go’ spectacle frame.

## Conclusion

Despite the many advantages of ready-made spectacles, it is important to identify which children have refractive error needs that cannot be met by ready-made spectacles; these children need custom-made spectacles made up by a dispensing optician (Table 1). Those who prescribe and dispense spectacles must be trained to be able to distinguish which type of spectacles would be suitable for each child.

**Figure 2:**
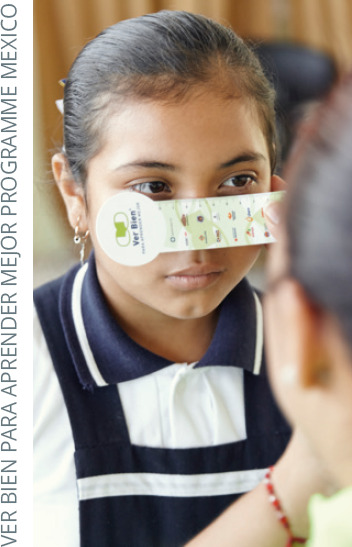
Measuring the inter-pupillary distance

Custom-made spectacles and ready-made spectacles should only be dispensed by a trained person, based on appropriate refractive technique, e.g., retinoscopy undertaken by a competent practitioner. All children who require spectacles must have their inter-pupillary distance measured to ensure the correct size spectacles are fitted (Figure 2).

**Table 1:** Indications for ready-made and custom-made spectacles

	Ready-made spectacles	Custom-made spectacles
Improvement in vision with spherical equivalent lenses	The same or only one line less than with full correction	Visual acuity with full correction is more than one line better than with the spherical equivalent
Difference in the spherical equivalent in right and left eyes	Not more than 1.00D	More than 1.00D
Astigmatism	Maximum of 0.75D cylinder in both eyes	More than 0.75D cylinder in one or both eyes
Maximum spherical equivalent	+ or −3.50D	No limit
Inter-pupillary distance between the eyes and the frames available	Not more than +/− 2 mm	This may be more than +/− 2 mm
Comfort of spectacle frames	As comfortable as custom made spectacles	
